# Supplementary material for: Increased nitrous oxide emissions from global lakes and reservoirs since the pre-industrial era
Source: Nat Commun. 2024 Jan 31;15:942. doi: 10.1038/s41467-024-45061-0 (PMC10830459; doi:10.1038/s41467-024-45061-0)
Supplement: Supplementary file 1 — Supplementary Information [file 41467_2024_45061_MOESM1_ESM.pdf]

Nature Communications

Supplementary Information for

Increased nitrous oxide emissions from global lakes and reservoirs since the pre-industrial era

Ya Li[a,b,c], Hanqin Tian[d*], Yuanzhi Yao[e], Hao Shi[a], Zihao Bian[f,b], Yu Shi[b,g], Siyuan Wang[a,c], Taylor Maavara[h], Ronny Lauerwald[i], Shufen Pan[j,b]

[a]State Key Laboratory of Urban and Regional Ecology, Research Center for Eco-Environmental Sciences, Chinese Academy of Sciences, Beijing 100085, China;
[b]College of Forestry, Wildlife and Environment, Auburn University, Auburn, AL 36832, USA;
[c]University of Chinese Academy of Sciences, Beijing 100049, China;
[d]Center for Earth System Science and Global Sustainability, Schiller Institute for Integrated Science and Society, Department of Earth and Environmental Sciences, Boston College, Chestnut Hill, MA 02467, USA;
[e]School of Geographic Sciences, East China Normal University, Shanghai 610000, China;
[f]School of Geography, Nanjing Normal University, Nanjing 210023, China;
[g]College of Urban and Environmental Sciences, Peking University, Beijing 100871, China;
[h]School of Geography, University of Leeds, UK;
[i]Université Paris-Saclay, INRAe/APT-UMR 1402 EcoSys, 22 Place de l'Agronomie, 91120 Palaiseau, France
[j]Center for Earth System Science and Global Sustainability, Department of Engineering, Boston College, Chestnut Hill, MA 02467, USA

[*] To whom correspondence should be addressed. Email: hanqin.tian@bc.edu

Supplementary Text 1: The simulation of advective N_2O flux F_a

In the sub-grid level, the inflow and outflow rates of river channels, lakes and reservoirs in the model can be described by the following equations:

$$\frac{\partial Q_{channel,out}}{\partial x}+L\frac{\partial h}{\partial t}=Q_{channel,\ in} \tag{1}$$

$$Q_{sublake,\ in}=Q_{hill}\times(Area_{upstream,\ sublake}\div Area_{grid}) \tag{2}$$

$$Q_{subreservoir,\ in}=Q_{hill}\times(Area_{upstream,\ subreservoir}\div Area_{grid}) \tag{3}$$

$$Q_{subnetwork,\ in}=Q_{hill}-Q_{sublake,\ in}-Q_{subreservoir,\ in}+Q_{sublake,\ out}+Q_{subreservoir,\ out} \tag{4}$$

Where $Q_{channel,out}$ and $Q_{channel,in}$ are outflow rates and inflow rates of river channels (note that the label "channel" can be hillslope, subnetwork, and main channel, respectively); L the length of the river channels; h is the depth of runoff; note that the outflow rates of river channels shown in the equation (1) are solved by Kinematic Wave Method in our model (1); $Q_{subnetwork,in}$, $Q_{sublake,in}$, and $Q_{subreservoir,in}$ are inflow rates of subnetworks, small lakes, and small reservoirs ($m^3\ d^{-1}$), respectively; $Q_{sublake,out}$ and $Q_{subreservoir,out}$ are outflow rates of small lakes and small reservoirs($m^3\ d^{-1}$), which are calculated from the water residence time of lakes and reservoirs obtained from HydroLAKES dataset; Q_{hill} are outflow rates of hillslope; $Area_{grid}$, $Area_{upstream,sublake}$, $Area_{upstream,subreservoir}$ are the areas of the grid cell, the upstream of small lakes and small reservoirs, respectively.

Accordingly, the advective N_2O fluxes through subnetworks ($F_{a,subnetwork}$), small lakes ($F_{a,sublake}$) and small reservoirs ($F_{a,subreservoir}$) are described as:

$$F_{a,sublake}=Q_{sublake,in}\times C_{hill}-Q_{sublake,out}\times C_{sublake,out} \tag{5}$$

$$F_{a,subreservoir}=Q_{subreservoir,in}\times C_{hill}-Q_{subreservoir,out}\times C_{subreservoir,out} \tag{6}$$

$$F_{a,subnetwork}=Q_{subnetwork,in}\times C_{hill}+Q_{sublake,out}\times C_{sublake,out}+Q_{subreservoir,out}\times C_{subreservoir,out}+Y_{g/h}-Q_{sunetwork,out}\times C_{subnetwork,out} \tag{7}$$

where $Q_{subnetwork,out}$ are inflow rates of subnetworks ($m^3\ d^{-1}$); C_{hill}, $C_{subnetwork,out}$, $C_{sublake,out}$, and $C_{subreservoir,out}$ are concentrations ($g\ N\ m^{-3}$) of dissolved N_2O in the outflow of hillslope (C_{hill} equals to the equilibrium N_2O concentration), subnetworks, small lakes, and small reservoirs, respectively; We assumed that the dissolved N_2O yield ($Y_{g/h}$ in $g\ N\ d^{-1}$) in groundwater and hyporheic zones is linearly related to the land nitrate (NO_3^-) leaching rate:

$$Y_{g/h}=\sum r_{g/h}\times Loading_{NO3-}\times Area_{veg} \tag{8}$$

where $r_{g/h}$ is the ratio of N_2O production over the leached NO_3^- (unitless), $Loading_{NO3-}$ is the NO_3^- leaching rate from land ($g\ N\ m^{-2}\ d^{-1}$) calculated in DLEM-TAC, and $Area_{veg}$ is vegetation area (m^2) of different plant functional types.

The advective N_2O fluxes through the main channels ($F_{a,main}$), large lakes ($F_{a,largelake}$), and large reservoirs ($F_{a,largereservoir}$) are described as:

$$F_{a,largelake} = Q_{up,out} \times C_{up,out} - Q_{largelake,out} \times C_{largelake,out} \quad (9)$$

$$F_{a,largereservoir} = Q_{largelake,out} \times C_{largelake,out} - Q_{largereservoir,out} \times C_{largereservoir,out} \quad (10)$$

$$F_{a,main} = Q_{largereservoir,out} \times C_{largereservoir,out} + Q_{sunetwork,out} \times C_{subnetwork,out} - Q_{main,out} \times C_{main,out} \quad (11)$$

where $Q_{up,out}$ and $Q_{main,out}$ are the outflow rates ($m^3\ d^{-1}$)of upstream grid cells and the main channels in the current grid cell, which are solved by Kinematic Wave Method (equation 1); $Q_{largelake,out}$ are outflow rate of large lakes ($m^3\ d^{-1}$), which are calculated from the water residence time of lakes obtained from the HydroLAKES dataset; $Q_{largereservoir,out}$ are outflow rates of large reservoirs ($m^3\ d^{-1}$), which are quantified through a dam operation module using natural flow, dam storage and dam type as model inputs (2, 3); $C_{up,out}$, $C_{main,out}$, $C_{largelake,out}$, $C_{largereservoirs,out}$ are the associate N_2O concentrations ($g\ N\ m^{-3}$).

Supplementary Text 2: The simulation of N_2O production within inland waters Y_{water}

Dissolved N_2O production (g N d^{-1}) in water column was calculated from both nitrification and denitrification:

$$Y_{water}=R_{nitrif}\times k_{nitrif}\times C_{nhx}\times Q+R_{denitrif}\times k_{denitrif}\times C_{noy}\times Q \tag{12}$$

where k_{nitrif} and $k_{denitrif}$ are the nitrogen (N) removal efficiency (unitless) through nitrification and denitrification, respectively; R_{nitrif} and $R_{denitrif}$ are the associated ratio of N_2O production through nitrification and denitrification, respectively (4). C_{nhx} and C_{noy} are N contents of the water (g N m^{-3}); Q is discharge (m^3 d^{-1}); The nitrification or denitrification efficiency (k) can be estimated as:

$$k= exp(\frac{-v}{\Delta d}) \tag{13}$$

where v is the settling velocity (m d^{-1}) of NO_3^- or ammonia (NH_4^+) through nitrification or denitrification, respectively, and Δd is the hydraulic load (m d^{-1}) for water flow into the downstream grid cell. v can be simulated by a first-order kinetics equation (5):

$$v= v_{ref}(Q_{10})^{\frac{T-T_s}{10}} \tag{14}$$

where v_{ref} is the settling velocity of NO_3^- or NH_4^+ at the reference temperature of 20 ˚C, Q_{10} is the change fraction of NO_3^- or NH_4^+ reaction rates at a temperature change of 10 ˚C and assigned 2.0 here, T is the water temperature (˚C), and T_s is the reference temperature (20 ˚C). Δd can be expressed as:

$$\Delta d=\frac{Q}{A_s} \tag{15}$$

where Q is discharge (m^3 d^{-1}), and A_s is the surface area of the waterbody.

Supplementary Text 3: The simulation of N_2O reduction R

The reduction of N_2O in inland waters is computed according to a first-order kinetics equation:

$$R=K_{reduction}M_{N2O} \tag{16}$$

where $K_{reduction}$ is the reduction rate (d^{-1}), and M_{N2O} is the content of dissolved N_2O (g N) in inland waters.

Supplementary Text 4: The simulation of N_2O efflux through the air-water interface E

The N_2O emission or sink in inland waters was estimated as:

$$E = K_{N2O} \times (C_{N2O} - C_{N2Oeq}) \times Area_{water} \tag{17}$$

where $Area_{water}$ is the water surface area (m^2), of which riverine area can be estimated based on channel geometry (6-8), the area of lakes and reservoirs are derived from the HydroLAKES and GRanD dataset. C_{N2O} and C_{N2Oeq} are dissolved N_2O concentration (mg L^{-1}) and atmospheric equilibrium N_2O concentration (mg L^{-1}) (9), respectively. K_{N2O} (m d^{-1}) is gas exchange rate (10).

Supplementary Table 1: Surface area of large and small lentic systems derived from the HydroLAKES and GRanD datasets.

	**Large lakes**	**Small lakes**	**Large reservoirs**	**Small reservoirs**
**Surface Area** (km^2)	1,442,678.05	1,029,106.46	399,361.80	29,390.70

Supplementary Table 2: Dynamic agricultural N_2O emission factor for inland waters (EF_{Ag}) from the 1920s.

	**1920s**	**1950s**	**1980s**	**1990s**	**2010s**
**N_2O emissions**ꜜ (Gg N yr^{-1})	1.3	10.8	92.6	112.2	137.0
**Ag-N addition**[§] (Tg N yr^{-1})	73.5	89.9	190.7	211.8	267.3
**EF_{Ag}** (%)	0.002	0.012	0.049	0.053	0.051

Note:

ꜜ means the N_2O emissions from inland waters induced by agricultural N addition, which calculated as the difference of inland water N_2O emissions between Simulation 1 and Simulation 6;

§ includes the N fertilizer and N manure application in the agriculture activities.

Supplementary Table 3: The national-scale agricultural N_2O emission factors for inland waters in the 2010s.

No.	Country name	EF_{Ag} (%)	No.	Country name	EF_{Ag} (%)
1	Croatia	0.3414	45	Chile	0.0383
2	Serbia	0.2390	46	Spain	0.0383
3	Montenegro	0.1991	47	Armenia	0.0374
4	Srilanka	0.1805	48	United States	0.0371
5	Finland	0.1804	49	Suriname	0.0352
6	Poland	0.1698	50	Azerbaijan	0.0348
7	Czech Republic	0.1602	51	Malawi	0.0330
8	India	0.1555	52	Honduras	0.0321
9	Slovakia	0.1542	53	Colombia	0.0309
10	France	0.1507	54	Thailand	0.0309
11	Germany	0.1449	55	Uzbekistan	0.0291
12	Hungary	0.1430	56	Togo	0.0288
13	Iraq	0.1190	57	Norway	0.0261
14	Nigeria	0.1185	58	Mali	0.0259
15	Canarias	0.1180	59	Morocco	0.0241
16	Pakistan	0.1160	60	Malaysia	0.0232
17	Slovenia	0.1118	61	Ecuador	0.0229
18	Turkey	0.1071	62	Syrian Arab Republic	0.0229
19	Belgium	0.1024	63	Peru	0.0225
20	Romania	0.1010	64	Guatemala	0.0215
21	El Salvador	0.1009	65	Reunion	0.0210
22	Luxembourg	0.0909	66	Georgia	0.0210
23	Portugal	0.0891	67	Venezuela	0.0209
24	Moldova	0.0855	68	Cambodia	0.0207
25	Democratic People's Republic of Korea	0.0847	69	Tajikistan	0.0191
26	Bulgaria	0.0791	70	Ghana	0.0190
27	Italy	0.0761	71	Costa Rica	0.0188
28	Greece	0.0761	72	Canada	0.0181
29	Russian Federation	0.0745	73	Brazil	0.0180
30	Austria	0.0685	74	Cote d'lvoire	0.0175
31	China	0.0673	75	Kazakhstan	0.0173
32	Iran	0.0666	76	Paraguay	0.0167
33	United Kingdom	0.0607	77	Cameroon	0.0158
34	Netherlands	0.0604	78	Turkmenistan	0.0155
35	Albania	0.0594	79	New Caledonia	0.0148
36	Sweden	0.0576	80	Viet Nam	0.0138
37	Ukraine	0.0526	81	Liberia	0.0135
38	Switzerland	0.0514	82	Republic of Korea	0.0129
39	Guyana	0.0503	83	Zimbabwe	0.0123
40	Indonesia	0.0496	84	Kyrgyzstan	0.0123
41	Philippines	0.0474	85	Mexico	0.0121
42	Bosnia and Herzegovina	0.0461	86	Gambia	0.0117
43	Bahamas	0.0420	87	Japan	0.0116
44	Bangladesh	0.0390	88	Nepal	0.0113

Supplementary Table 3 Continued

No.	Country name	EF_{Ag} (%)	No.	Country name	EF_{Ag} (%)
**89**	Belarus	0.0111	**122**	Senegal	0.0027
**90**	Papua New Guinea	0.0101	**123**	Guinea-Bissau	0.0027
**91**	Zambia	0.0099	**124**	Tanzania	0.0024
**92**	Sudan	0.0098	**125**	Australia	0.0022
**93**	Laos	0.0098	**126**	Burundi	0.0022
**94**	Mozambique	0.0095	**127**	Puerto Rico	0.0021
**95**	Argentina	0.0093	**128**	Ethiopia	0.0020
**96**	Tunisia	0.0090	**129**	Gabon	0.0020
**97**	Jamaica	0.0088	**130**	Ireland	0.0020
**98**	Estonia	0.0088	**131**	Uganda	0.0019
**99**	Chad	0.0087	**132**	Democratic Republic of Congo	0.0019
**100**	Swaziland	0.0085	**133**	Benin	0.0018
**101**	Niger	0.0082	**134**	Israel	0.0018
**102**	Haiti	0.0079	**135**	Congo	0.0017
**103**	Denmark	0.0079	**136**	Uruguay	0.0016
**104**	Nicaragua	0.0077	**137**	Mauritania	0.0014
**105**	Lebanon	0.0075	**138**	Guinea	0.0014
**106**	Latvia	0.0075	**139**	Botswana	0.0014
**107**	Afghanistan	0.0073	**140**	Brunei	0.0014
**108**	Fiji	0.0073	**141**	Mongolia	0.0013
**109**	Lithuania	0.0072	**142**	Somalia	0.0012
**110**	Caspian Sea	0.0069	**143**	Libya	0.0010
**111**	South Africa	0.0067	**144**	Angola	0.0010
**112**	Kenya	0.0063	**145**	Cyprus	0.0009
**113**	Algeria	0.0058	**146**	New Zealand	0.0007
**114**	Equatorial Guinea	0.0056	**147**	Namibia	0.0007
**115**	Burkina Faso	0.0055	**148**	Belize	0.0003
**116**	East Timor	0.0044	**149**	Eritrea	0.0002
**117**	Myanmar	0.0039	**150**	Sierra Leone	0.0002
**118**	Panama	0.0038	**151**	Kuwait	0.0002
**119**	Bhutan	0.0035	**152**	Yemen	0.0002
**120**	Rwanda	0.0029	**153**	Central African Republic	0.0001
**121**	Lesotho	0.0028	**154**	Madagascar	0.0001

Supplementary Table 4: The comparison on N_2O emissions from lentic systems (the GWP for N_2O is assigned values of 273).

	Methods	**N_2O emissions (Gg N yr^{-1})**	**CO_2eq. (Tg CO2eq yr^{-1})**	References
**Lakes**				
	Processed-based	56.8	24.4	This study
	Meta analysis	160.0-380.0	68.6-163.0	(11)
	Meta analysis	330.9	142.0	(12)
	Data synthesis	76.4	32.8	(13)
	Mechanistic model	29.4	12.6	(14)
	Mechanistic model	80.0	34.3	(15)
**Reservoirs**				
	Processed-based	7.8	3.3	This study
	Meta analysis	70.0	30.0	(12)
	Data synthesis	30.0	12.9	(16)
	Data synthesis	26.1	11.2	(13)
	Mechanistic model	33.6	14.4	(14)
	Mechanistic model	52.9	22.7	(17)
	Mechanistic model	450.0	193.1	(15)
**Total**				
	Processed-based	64.6	27.7	This study
	Meta analysis	400.9	172.0	(12)
	Meta analysis	583.0	250.1	(18)
	Data synthesis	102.5	44.0	(13)
	Mechanistic model	63.0	27.0	(14)
	Mechanistic model	530.0	227.4	(15)

Supplementary Table 5: The emission factors of N_2O emissions from inland waters published in the literatures.

**Original**				
Regions	*Types*	*Relative to*	*EFs %*	*References*
Global	Inland waters	nitrate concentration	0.25	(19)
Global	Inland waters	N loads	0.75	(4)
Global	Inland waters	nitrate concentration	0.26	(20)
Global	Inland waters⸸	nitrate concentration	0.83	(12)
Global	River and Reservoir	N loads	0.18-0.45	(17)
**Discounted based on a 24% loss of agricultural N addition through runoff and leaching, as recommended by the IPCC report (19)**				
Regions	*Types*		*EFs %*	*References*
Global	Inland waters		0.062	(19)
Global	Inland waters		0.225[§]	(4)
Global	Inland waters		0.062	(20)
Global	Inland waters		0.199	(12)
Global	River and Reservoir		0.043-0.108	(17)

Note:

⸸ The types of water systems include streams, rivers, lakes, and reservoirs.

§ the value is discounted by the loss fraction of 30% as assumed in that study.

Supplementary Table 6: Key parameters in the DLEM-TAC aquatic N_2O module for the simulation on lentic systems.

Parameter	Description	Value	Ranges	Unit	Ref.
$\boldsymbol{Vf_{NO3l}}$	NO_3^- uptake velocity in lakes	3E-08	1.90E-08-8.37E-07	m/s	(21, 22)
$\boldsymbol{Vf_{NH4l}}$	NH_4^+ uptake velocity in lakes	2E-07	1.90E-08-8.37E-07	m/s	(21, 22)
$\boldsymbol{Vf_{NO2l}}$	NO_2 uptake velocity in lakes	2E-07	1.90E-08-8.37E-07	m/s	(21, 22)
$\boldsymbol{Vf_{NO3r}}$	NO_3^- uptake velocity in reservoirs	2E-06	3.17E-10-2.57E-06	m/s	(21)
$\boldsymbol{Vf_{NH4r}}$	NH_4^+ uptake velocity in reservoirs	2E-06	3.17E-10-2.57E-06	m/s	(21)
$\boldsymbol{Vf_{NO2r}}$	NO_2 uptake velocity in reservoirs	2E-06	3.17E-10-2.57E-06	m/s	(21)
$\boldsymbol{R_{denitrif}}$	Ratio of N_2O production from denitrification	1%	0.87-9.52%	unitless	(23)
$\boldsymbol{R_{nitrif}}$	Ratio of N_2O production from nitrification	1%	0.3-25%	unitless	(24, 25)
$\boldsymbol{K_{reduction}}$	N_2O consumption rate	0.012	0.0057~0.0344	1/d	(26, 27)⸸

Note:

⸸ The value was introduced from Yao et al. (2019) which convert from the areal N_2O consumption rate according to the study by Kolb et al. (2012). The areal N_2O consumption rate, as reported by Kolb et al. (2012), ranged from −9.4 to −56.8 nmol m^{-2} h^{-1}. Yao et al. (2019) assumed the depth of the water column (bogs) as 6-m, the waterbody consumed N_2O to keep the dissolved N_2O concentration equal to the atmospheric equilibrium N_2O concentration (6.6 nmol L^{-1}). Thus, the N_2O consumption rate is converted as: -9.4(56.8) nmol m^{-2} h^{-1} × 24 hr / 6-m / 1000 / 6.6 nmol L^{-1} = 0.0057(0.0344) 1/d.

Supplementary Table 7: Information of observed data for N_2O emissions from inland waters.

**N_2O emissions from lakes**						
Name	*Region*	*Year*	*Land use*	*Collected method*	*Emissions mg N m^{-2} yr^{-1}*	*Literature source*
Tanganyika	AF	2019-2021	Forest	Calculated[ǂ]	-4.14	(28)
Albert	AF	2019	Cropland	Calculated	7.67	(28)
Edward	AF	2016-2019	Cropland	Calculated	5.62	(28)
Mai Ndombe	AF	2015	Forest	Calculated	61.83	(28)
Tumba	AF	2014	Forest	Calculated	27.59	(28)
Kamohonjo	AF	2019	Grassland	Calculated	2.45	(28)
Yandja	AF	2010	Forest	Calculated	-0.66	(28)
Postilampi	EU	1996-1998	Forest	SC[§]	12.57	(29)
Vehmasjarvi	EU	1997-1998	Forest	SC	14.74	(29)
Jankalaisenlampi	EU	1994	Peatland	SC	1.74	(29)
Kuivajärvi	EU	2011-2012	Peatland	Calculated	81.02	(30)
Taihu	AS	2003-2004	Cropland	Calculated	275.66	(31)
Baiyangdian	AS	2011		SC	249.54	(32)
Huahu	AS	2009	Meadow	SC	25.09	(33)
Chaohu	AS	2016-2017	Cropland	Calculated	211.12	(34)
Poyang	AS	2004	Forest	SC	58.53	(35)
Lagoon Bamenwan	AS	2008		Calculated	136.44	(36)
Lagoon Bo'ao	AS	2008		Calculated	43.96	(36)
Gahai	AS	2011-2012			119.85	(37)
Wivenhoe	OCE	2011-2012	Grassland	Calculated	19.74	(38)
Baroon	OCE	2011-2012	Grassland	Calculated	10.45	(38)
Okaro	OCE			Calculated	3.07	(39)
Superior	NA	2015	Forest	Calculated	7.21	(40)
Erie	NA	2015	Urban	Calculated	47.32	(40)
Long Lake	NA	2010-2013	Forest	SC	2.60	(41)
Muskegon	NA	2012-2013	Forest	SC	108.68	(42)
Shoal	NA	2002	Cropland	SC	1.16	(43)
Slocan	NA	2002			24.39	(43)
Trout	NA	2002			-3.48	(43)
Lakes in SM 2region	NA	2001-2003		FC[÷]	18.58	(43)
Lakes in Manic 5region	NA	1999-2002		FC	11.61	(43)
Lakes in Opinaca region	NA	2003		FC	15.1	(43)
Lakes in RB region	NA	1999-2002		FC	8.12	(43)
Lakes in La Grande 3region	NA	2003		FC	16.26	(43)
Lakes in La Grande 4region	NA	2003		FC	-26.71	(43)
Lakes in Laforge 1region	NA	1995-2003		FC	3.47	(43)
Lakes in Caniapiscau region	NA	2003		FC	3.47	(43)
Lakes in Bersimis region	NA	2002		FC	13.95	(43)
Lakes in Saguenay region	NA	2011-2012	Forest	Calculated	62.34	(44)
Lakes in Schefferville region	NA	2011-2012	Forest	Calculated	16.35	(44)

Lakes in Côte Nord region	NA	2011-2012	Forest	Calculated	-17.37	(44)
**N_2O emissions from reservoirs**						
Name	*Region*	*Year*	*Land use*	*Collected method*	*Emissions mg N m^{-2} yr^{-1}*	*Literature source*
Masinga	AF	2011-2013		Calculated	5.11	(45)
Lokka	EU	1994-1995		Calculated	6.03	(46)
Porttipahta	EU	1995		Calculated	13.54	(46)
Eguzon	EU	2011	Cropland	Calculated	90.45	(47)
Xiaowan	AS	2019		Calculated	91.57	(48)
Siling	AS	2017		Calculated	67.71	(49)
Hongfeng	AS	2017		Calculated	87.64	(49)
Yudushan	AS	2009-2010		SC	278.72	(50)
Dingjie	AS	2012	Urban	SC	142.15	(51)
Xi'anjiang	AS	2014-2016		SC	85.97	(52)
Hedi	AS	2011		Calculated	19.45	(53)
Jinjiang	AS	2011		Calculated	8.53	(53)
Qieyeshi	AS	2011		Calculated	35.65	(53)
Songmushan	AS	2011		Calculated	33.06	(53)
Xinfengjiang	AS	2011		Calculated	14.00	(53)
Changhu	AS	2011		Calculated	47.78	(53)
Fengshuba	AS	2011		Calculated	22.43	(53)
Jurong	AS	2010-2012	Cropland	Calculated	21.99	(54)
Xipi	AS	2012-2013	Forest	Calculated	233.78	(55)
dam in Jiulong River	AS	2013-2014	Forest	SC	241.69	(56)
Xiaolangdi	AS	2017	Cropland	Calculated	68.73	(57)
Three Gorges Dam	AS	2010-2012		SC	416.82	(58)
reservoir in southwest China	AS	2014-2015	Cropland	Calculated	741.29	(59)
Gold Creek	OCE	2012-2014	Forest	SC	14.77	(60)
Eagle Creek	NA	2005-2008	Cropland	Calculated	243.89	(61)
Baskatong	NA	2002		FC	3.48	(43)
Manic 1	NA	1999-2003		FC	26.72	(43)
La Grande 3	NA	2003		FC	9.29	(43)
Laforge 1	NA	1993-2003		FC	-12.79	(43)
Caniapiscau	NA	2003		FC	5.83	(43)
Cabonga	NA	1996-2002		FC	23.21	(43)
Robert-Bourassa	NA	1997-2001		FC	16.25	(43)
Robertson	NA	2001-2003		FC	15.07	(43)
Bersimis	NA	2002-2003		FC	9.29	(43)
Toulnoustoucna	NA	2002-2003		FC	11.56	(43)
Fortuna	SA	2003		FC	35.77	(62)
Serra de Mesa	SA	2003-2004	Grassland	Calculated	15.33	(63)
Tres Marias	SA	2011-2013	Grassland	FC	40.21	(64)
Xingo	SA	2011-2013	Forest	FC	58.08	(64)
Funil	SA	2011-2013	Forest	FC	87.07	(64)
Segredo	SA	2011-2013	Forest	FC	100.39	(64)
Itaipu	SA	2011-2013	Forest	FC	61.93	(64)
**N_2O emissions from streams and rivers**						

Name	*Region*	*Year*	*Land use*	*Collected method*	*Emissions mg N m^{-2} yr^{-1}*	*Literature source*
Congo	AF	2010-2014	Forest	Calculated	86.87	(65)
Ogooue	AF	2012-2014	Forest	Calculated	104.76	(65)
Niger	AF	2011-2013	Cropland	Calculated	23	(65)
Zambezi	AF	2012-2013	Shrubland	Calculated	10.22	(65)
AGS	AF	2011-2013	Grassland	Calculated	89.43	(65)
stream in Sweden	EU	2014-2015	Cropland	Calculated	1242.17	(66)
River Orwell	EU	2001-2002		Calculated	235.38	(67)
River Conwy	EU	2001-2002		Calculated	89.27	(67)
West River of Jiulong River	AS	2011	Forest	Calculated	55.7	(68)
North River of Jiulong River	AS	2011	Forest	Calculated	81.76	(68)
Yellow river	AS	2017	Grassland	Calculated	35.77	(57)
Ashburton River	OCE	2007		Calculated	201.48	(69)
Ohio river	NA	2008-2009	Forest	FC	140.16	(70)
Hudson river	NA	1998-1999	Forest	Calculated	72.71	(71)
Grand river	NA	2007-2009	Cropland	Calculated	788.4	(72)
Plate river	NA	1994-1995	Urban	SC	551.88	(73)
Neuse river	NA	2001-2002	Forest	SC	560.64	(74)
NC_ag	NA	2003-2006	Cropland	Calculated	448.51	(75)
MI_ag	NA	2003-2006	Cropland	Calculated	45.41	(75)
MA_ag	NA	2003-2006	Cropland	Calculated	116.07	(75)
KS_ref_1	NA	2003-2006	Grassland	Calculated	1223.99	(75)
PR	NA	2003-2006	Forest	Calculated	406.46	(75)
Amazon mainstem	SA	1982-1985	Forest	Calculated	100	(76)

Note:

⸸ means the N_2O emission was calculated from measured concentration of dissolved N_2O;

δ means the N_2O emission was measured by static chambers;

⁘ means the N_2O emission was measured by floating chambers.

Supplementary Table 8: Information of observed data for nitrate concentration in inland waters.

**Nitrate concentrations in lakes**						
Name	*Region*	*Year*	*Land use*	*Collected depth*	*Conc. µg NO_3-N L^{-1}*	*Literature source*
Lake Kivu	AF	2012-2013	Forest	0-70 m	53.63	(77)
Lake Neusiedl	EU	2011-2012		0.1 m	167	(78)
Kuivajärvi	EU	2011-2012	Peatland	0.1-12 m	58.5	(30)
Stavsvatn lake	EU	1998	Pasture	0-1 m	56	(79)
Lochnagar lake	EU	1998	Shrubland	0-1 m	224	(79)
Paione Superiore	EU	1998	Grassland	0-1 m	392	(79)
Lago di Latte	EU	1998	Pasture	0-1 m	210	(79)
Schwarzsee ob Sölden	EU	1998		0-1 m	98	(79)
Red´o	EU	1998		0-1 m	154	(79)
Laghetto Inferiore	EU	1998	Grassland	0-1 m	224	(79)
Taihu	AS	2011-2016	Cropland	0.2 m	518	(80)
Poyang Lake	AS	2010		0-0.15 m	475	(81)
Upper Lake	AS	2003-2004		0-6 m	93	(82)
Lake Mikata	AS	2004-2005		0.5 m	106	(83)
Chaohu Lake	AS	2015-2017		Surface	1258.6	(84)
Baiyangdian Lake	AS	2008-2016			4022	(85)
West Lake	AS	2013	Forest	0.5 m	1460	(86)
Hongfeng Lake	AS	2013		0-25 m	1280	(87)
Xingkai Lake	AS	2007-2009		Surface	2170	(88)
Saguenay region	NA	2011-2012	Forest		11.2	(44)
Schefferville region	NA	2011-2012	Forest		1.9	(44)
Côte Nord region	NA	2011-2012	Forest		5.5	(44)
Upper Mystic Lake	NA	2004		0-20 m	645.96	(89)
**Nitrate concentrations in reservoirs**						
Name	*Region*	*Year*	*Land use*	*Collected depth*	*Conc. µg NO_3-N L^{-1}*	*Literature source*
Masinga	AF	2011-2013		0.5 m	55.16	(45)
Kamburu	AF	2011-2013		0.5 m	54.6	(45)
Gitaru	AF	2011-2013		0.5 m	73.92	(45)
Lokka	EU	1994-1995	Peatland	0.5-1 m	29.83	(46)
Porttipahta	EU	1995	Forest	0.5-1 m	134	(46)
Klimkowka	EU	2004-2013			3660	(90)
Czorsztyn	EU	2004-2013			2960	(90)
Dobczyce	EU	2004-2013			4110	(90)
reservoirs of the Wujiang River	AS	2017		0-60 m	563.36	(49)
Bukit Merah	AS	2016-2017	Forest	0.1-0.3 m	100	(91)
Hongjiadu	AS	2017		0.5-60 m	3900	(92)
Dongfeng	AS	2017		0.5-60 m	3600	(92)
Wujiangdu	AS	2017		0.5-60 m	3500	(92)
Guanting	AS	2005		Surface	2267	(93)
Gold Creek	OCE	2012-2014	Forest	0.2 m	8.4	(60)
William H. Harsha	NA	2012	Cropland	0.1-1 m	445.38	(94)
Eagle Creek	NA	2005-2008	Cropland	0.2-2.5 m	1400	(61)
Falls Lake	NA	2001-2002	Forest	0.1 m	57.57	(74)
Tuttle Creek	NA	1972-2010			2800	(95)

Kanopolis	NA	1972-2010			1100	(95)
**Nitrate concentrations in streams and rivers**						
Name	*Region*	*Year*	*Land use*	*Collected depth*	*Conc. μg NO_3-N L^{-1}*	*Literature source*
Tana	AF	2011-2013		0.5 m	71.26	(45)
1-3th rivers in Mara River	AF	2017-2019	Mixed		1050	(96)
Po (IT01052050)	EU	2008			1191.25	(97)
Po (IT01052050)	EU	2007			4006.91	(97)
Meuse (NL94_KEIZVR)	EU	2007			3283.84	(97)
river in Albania (AL047)	EU	2008			413.36	(97)
Danube (RO100100)	EU	2007			922.67	(97)
Vuoksi (FISW_1447)	EU	2002			1814.35	(97)
Adyar	AS	2003-2004		0.2 m	396.06	(98)
Tuojia	AS	2013-2016	Cropland		1660	(99)
Yangtze 1	AS	2006-2011	Forest	0.5 m	1450	(100)
Yangtze 2	AS	2006-2011	Forest	0.5 m	1740	(100)
Yangtze 3	AS	2006-2011	Forest	0.5 m	860	(100)
Yangtze 4	AS	2006-2011	Forest	0.5 m	1940	(100)
Yangtze 5	AS	2006-2011	Forest	0.5 m	1560	(100)
Yangtze 7	AS	2006-2011	Forest	0.5 m	3090	(100)
Nanfei	AS	2006-2011	Urban	0.5 m	514	(100)
Hangbu	AS	2006-2011	Cropland	0.5 m	732	(100)
West Jiulong	AS	2006-2011	Forest	0.5 m	2020	(100)
Heilong	AS	2007-2009		Surface	2057.5	(88)
Stream Castle	OCE		Pasture		30	(101)
Stream Harrys	OCE		Pasture		20	(101)
rivers in Schefferville region	NA	2011-2012	Forest		6.9	(44)
rivers in Côte Nord region	NA	2011-2012	Forest		25.7	(44)
Stream in Eagle Creek	NA	2005-2008	Cropland	0.2-2.5 m	3100	(61)
Stream in New Hampshire	NA	2010	Forest	Surface	710	(102)
Neuse	NA	2001-2002	Forest	0.1 m	346.27	(74)

Supplementary Table 9: Information of observed data for nitrogen loads from land.

Land use	***Lat (°N)***	***Lon (°E)***	***Region***	***Year***	***Type***	***Intensity (Kg N ha^{-1} yr^{-1})***	***Literature source***
Cropland	60.817	23.467	EU	1994-2001	TN	14	(103)
Cropland	25.5	-80.5	NA		TN	148.5	(104)
Cropland	28.256	116.925	AS		DON	3.8	(105)
Cropland	42.396	-85.375	NA	2009-2015	DON	2.3	(106)
Cropland	48.05	-4.067	EU	2007	NO_3^-	71	(107)
Cropland	48.317	2.383	EU	2007	NO_3^-	25	(107)
Cropland	48.933	4.233	EU	2003; 2007	NO_3^-	32	(107)
Cropland	-10	-50	SA	2014-2015	NO_3^-	89.7	(108)
Cropland	42.396	-85.375	NA	2009-2015	NO_3^-	17.37	(106)
Forest	23.156	112.511	AS	2009-2012	TDN	18	(109)
Forest	24.917	118.917	AS	1993	TDN	1.87	(110)
Forest	21.933	101.267	AS	1999	TDN	5.95	(111)
Forest	29.567	101	AS	2015	TDN	6.5	(112)
Forest	29.583	95.417	AS	2001	TDN	0.052	(113)
Forest	26.747	115.070	AS	1999-2000	TDN	3.074	(114)
Forest	28.399	113.296	AS		TDN	3.51	(115)
Forest	23.167	112.167	AS	2004-2005	DON	10.6	(116)
Forest	42.396	-85.375	NA	2009-2015	DON	1.6	(106)
Forest	41.833	-73.75	NA	2002-2003	DON	1.7	(117)
Forest	-3.25	-59.97	SA	1998-1999	NO_3^-	1.1	(118)
Forest	23.167	112.33	AS	2005	NO_3^-	9.5	(119)
Forest	23.067	113.033	AS	1998-1999	NO_3^-	7.89	(120)
Forest	23.3	113.533	AS	1998-1999	NO_3^-	6.83	(120)
Forest	26.883	106.85	AS		NO_3^-	1.55	(121)
Forest	26.833	109.75	AS	1995-2000	NO_3^-	1.74	(122)
Forest	141.6	40.667	AS	1990-1993	NO_3^-	9.1	(123)
Forest	42.396	-85.375	NA	2009-2015	NO_3^-	7.2	(106)
Forest	41.833	-73.75	NA	2002-2003	NO_3^-	4.2	(117)
Forest	18.5	-66.333	NA	2001-2002	NO_3^-	7.67	(124)
Forest	-3.25	-59.97	SA	1998-1999	NH_4^+	2.2	(118)
Forest	23.167	112.167	AS	2004-2005	NH_4^+	1.14	(116)
Forest	23.067	113.033	AS	1998-1999	NH_4^+	3.48	(120)
Forest	23.3	113.533	AS	1998-1999	NH_4^+	5.24	(120)
Forest	26.883	106.85	AS		NH_4^+	0.31	(121)
Forest	26.833	109.75	AS	1995-2000	NH_4^+	0.64	(122)
Forest	18.5	-66.333	NA	2001-2002	NH_4^+	2.19	(124)
Forest	41.833	-73.75	NA	2002-2003	NH_4^+	2.9	(117)
Grassland	-40.3	175.833	OCE	2005	TDN	3.23	(125)
Grassland	-40.3	175.833	OCE	2005	DON	2.37	(125)
Grassland	-40.3	175.833	OCE	2005	NO_3^-	0.77	(125)
Grassland	-37.779	175.315	OCE	2011-2012	NH_4^+	0.18	(126)
Grassland	53.917	9.917	EU	2001-2002	NH_4^+	0.1	(127)
Peatland	61.933	23.433	EU		TN	2.76	(128)
Peatland	65.85	29.14	EU	1992-1994	TN	1	(129)
Peatland	65.267	28.38	EU	1992-1994	TN	0.7	(130)
Peatland	63.883	28.794	EU	1992-1995	TN	1.8	(130)

Peatland	63.867	29.32	EU	1979-1995	TN	1.21	(130)
Peatland	60.3	24.45	EU		TN	2.26	(128)
Peatland	53.393	-1.907	EU	2012-2013	TDN	14.1	(129)
Peatland	53.393	-1.907	EU	2012-2013	DON	7.79	(129)
Peatland	65.267	28.38	EU	1992-1994	NO_3^-	0.11	(130)
Peatland	65.85	29.14	EU	1992-1994	NO_3^-	0.08	(130)
Peatland	62.167	22.491	EU	1971-1994	NO_3^-	0.02	(130)
Peatland	61.933	23.433	EU		NO_3^-	0.04	(128)
Peatland	63.883	28.794	EU	1992-1995	NO_3^-	0.06	(130)
Peatland	60.417	24.556	EU	1963-1994	NO_3^-	1.2	(130)
Peatland	62.167	22.491	EU	1971-1994	NH_4^+	0.14	(130)
Peatland	61.933	23.433	EU		NH_4^+	0.08	(128)
Peatland	65.85	29.14	EU	1992-1994	NH_4^+	0.02	(130)
Peatland	65.267	28.38	EU	1992-1994	NH_4^+	0.01	(130)

Supplementary Table 10: The experimental design for the simulation of N_2O emissions from lentic systems (all-combined simulation (simulation 1) and a series of factorial experiments were designed to attribute the contributions of climate (simulation 2), land use (simulation 3), atmospheric CO_2 concentration (simulation 4), N deposition (simulation 5) and agricultural N addition (simulation 6)).

	**Climate**	**Land use**	**CO_2**	**N deposition**	**Ag-N addition**
**Simulation 1**	1850-2019	1850-2019	1850-2019	1860-2019	1860-2019
**Simulation 2**	1850	1850-2019	1850-2019	1860-2019	1860-2019
**Simulation 3**	1850-2019	1850	1850-2019	1860-2019	1860-2019
**Simulation 4**	1850-2019	1850-2019	1850	1860-2019	1860-2019
**Simulation 5**	1850-2019	1850-2019	1850-2019	1860	1860-2019
**Simulation 6**	1850-2019	1850-2019	1850-2019	1860-2019	1860

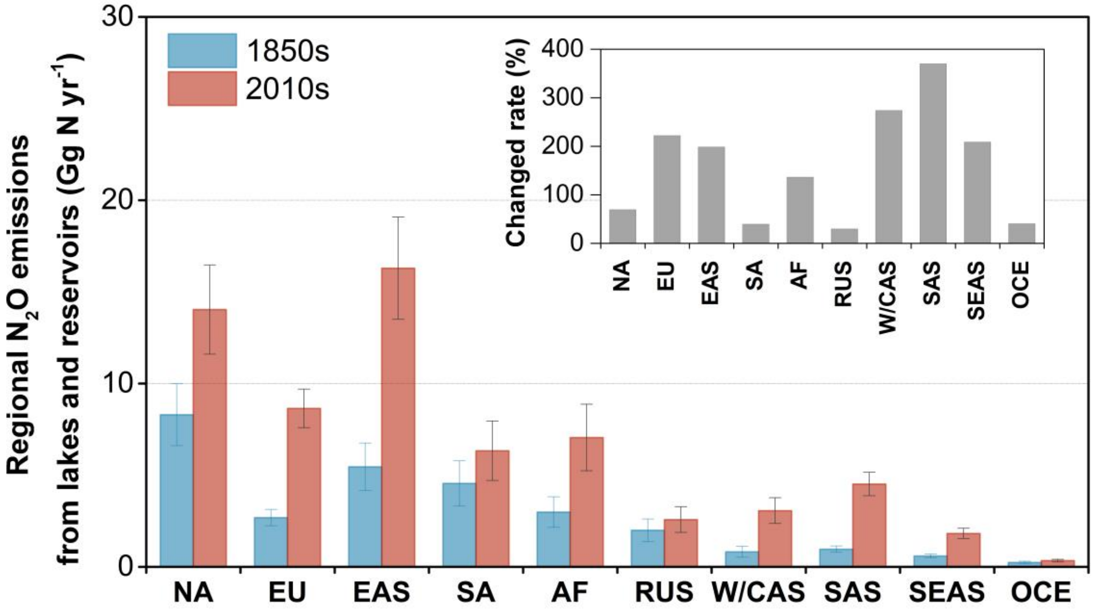

Supplementary Fig. 1: Comparison of regional mean N_2O emissions from lakes and reservoirs between the preindustrial period (the 1850s) and the recent decade (the 2010s) (NA: North America; SA: South America; EU: Europe; RUS: Russia; AF: Africa; SAS: South Asia; EAS: East Asia; SEAS: Southeast Asia; W/CAS: West/Central Asia; OCE: Oceania). The inserted figures are the changed rates of N_2O emissions from lakes and reservoirs between the two periods. Error bars denote the ±1 standard deviation in the period.

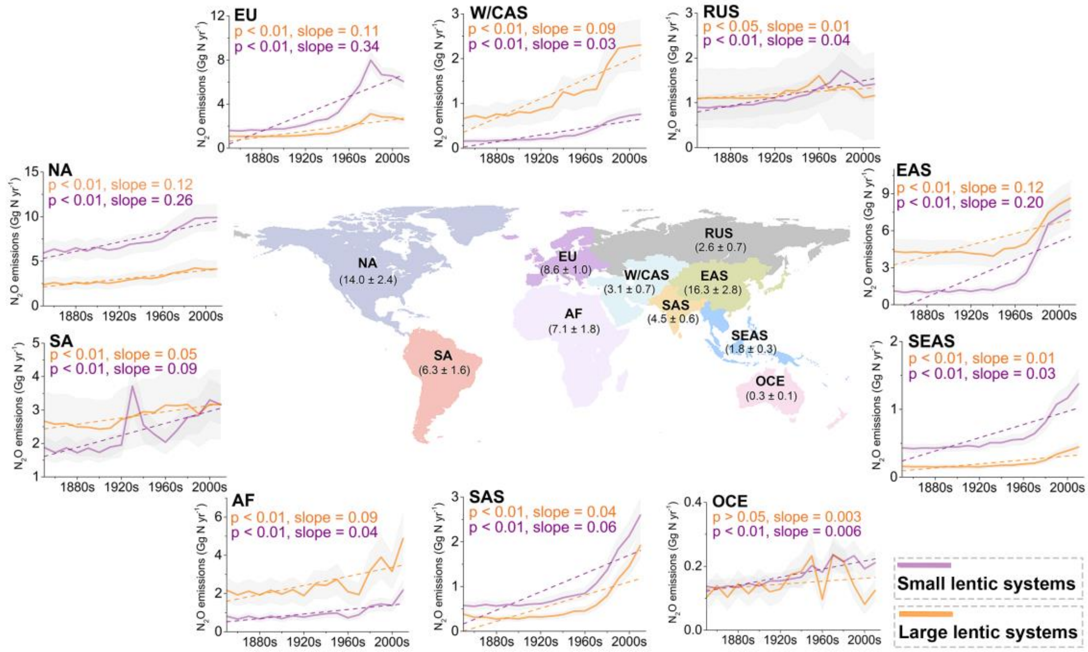

Supplementary Fig. 2: The long-term changes of regional N_2O emissions from large and small lentic systems (NA: North America; SA: South America; EU: Europe; RUS: Russia; AF: Africa; SAS: South Asia; EAS: East Asia; SEAS: Southeast Asia; W/CAS: West/Central Asia; OCE: Oceania). The map was drawn using the ArcMap v10.8.

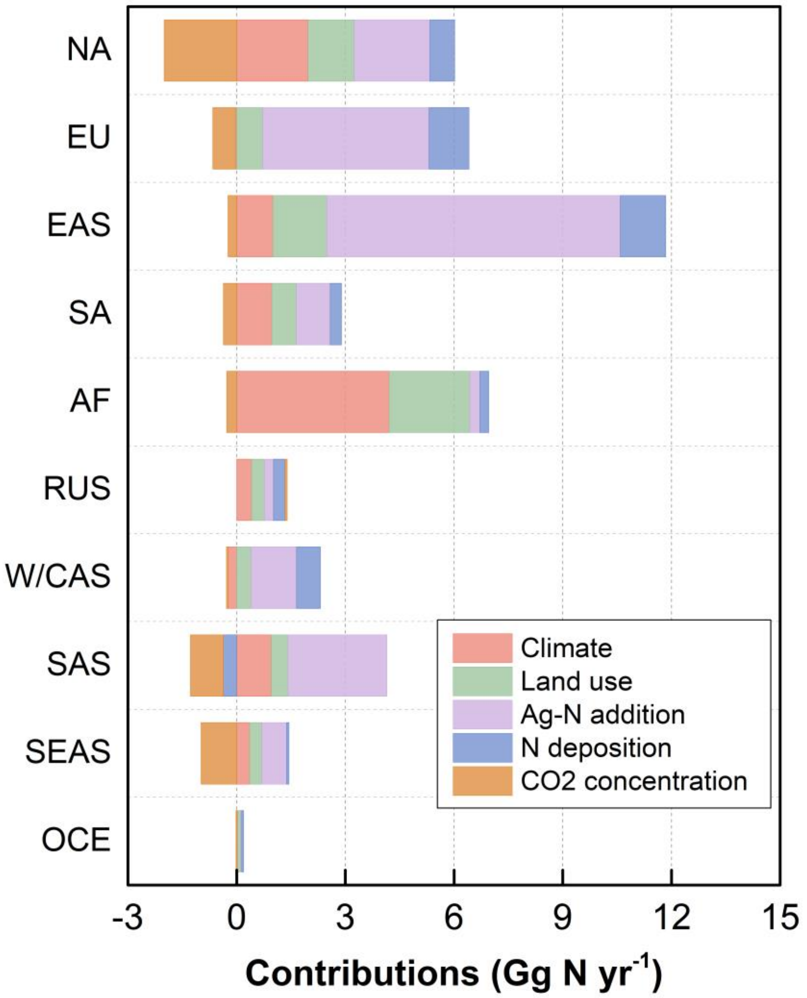

Supplementary Fig. 3: The contributions of environmental factors to changes in regional N_2O emissions from lakes and reservoirs in the 2010s (NA: North America; SA: South America; EU: Europe; RUS: Russia; AF: Africa; SAS: South Asia; EAS: East Asia; SEAS: Southeast Asia; W/CAS: West/Central Asia; OCE: Oceania). Ag-N addition includes nitrogen fertilizer and manure application.

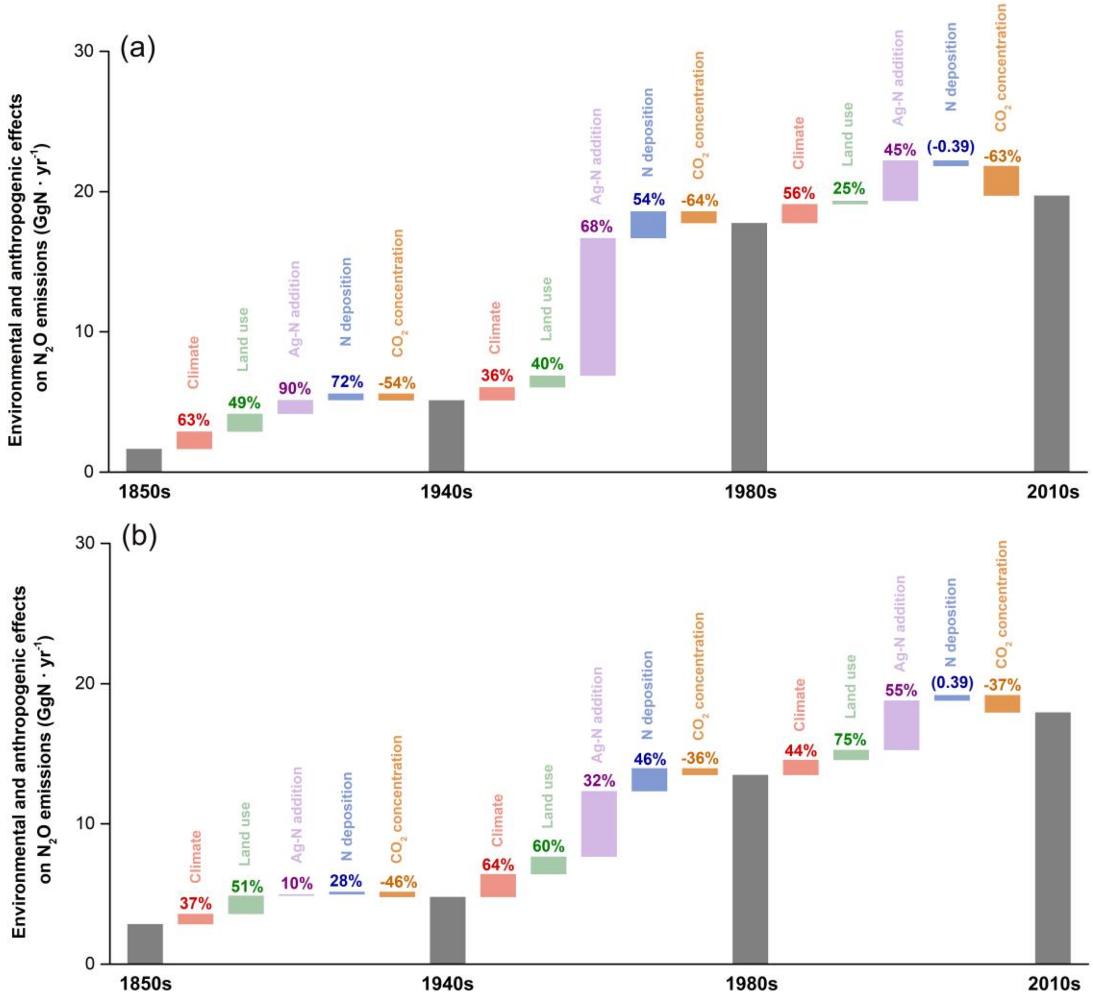

Supplementary Fig. 4: The relative contributions of environmental and anthropogenic factors to N_2O emission changes from small or large lakes and reservoirs over different time periods. (a) The relative contributions of environmental and anthropogenic factors to N_2O emission changes from small lentic systems. (b) The relative contributions of environmental and anthropogenic factors to N_2O emission changes from large lentic systems. The grey bars show mean decadal N_2O emissions induced by five forcing factors. The colored bars and their percentages represent the relative contribution of each forcing factor to the net change of total effect for the corresponding periods. Ag-N addition represents the agricultural nitrogen additions, which includes synthetic fertilizer and manure application.

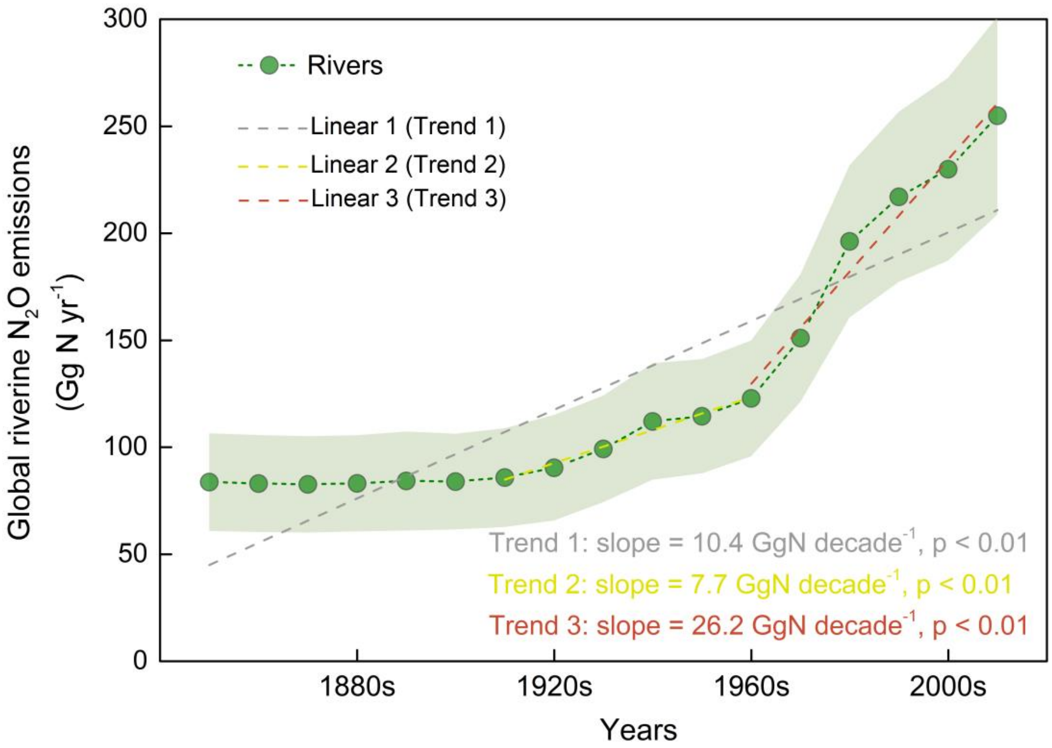

Supplementary Fig. 5: Dynamic N_2O emissions from global streams and rivers during the 1850s-2010s.

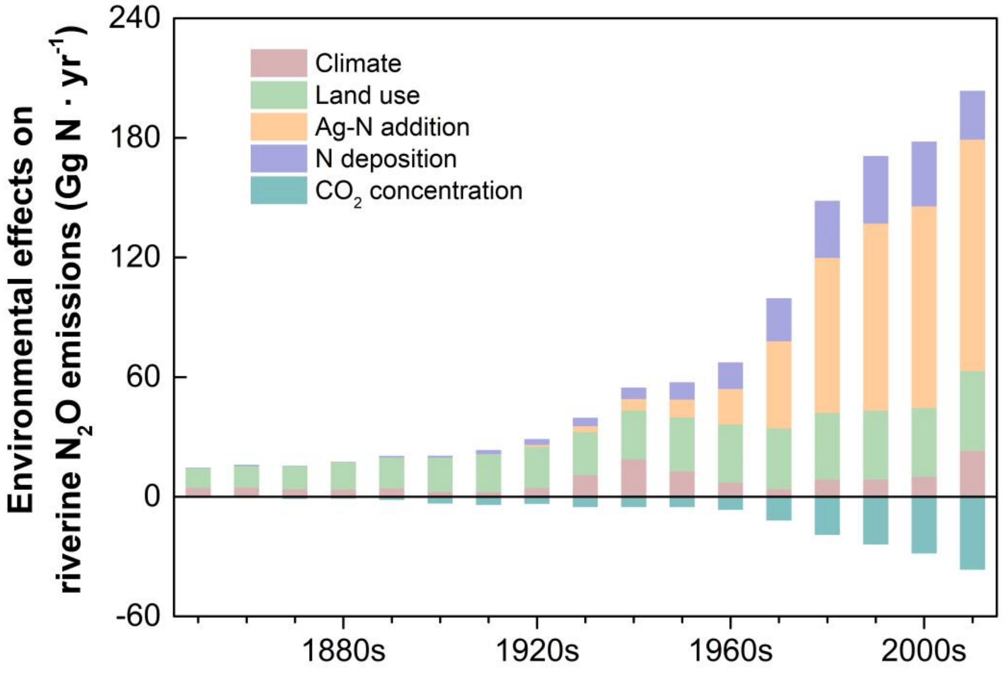

Supplementary Fig. 6: The effects of environmental factors on global riverine N_2O emissions from the 1850s to the 2010s.

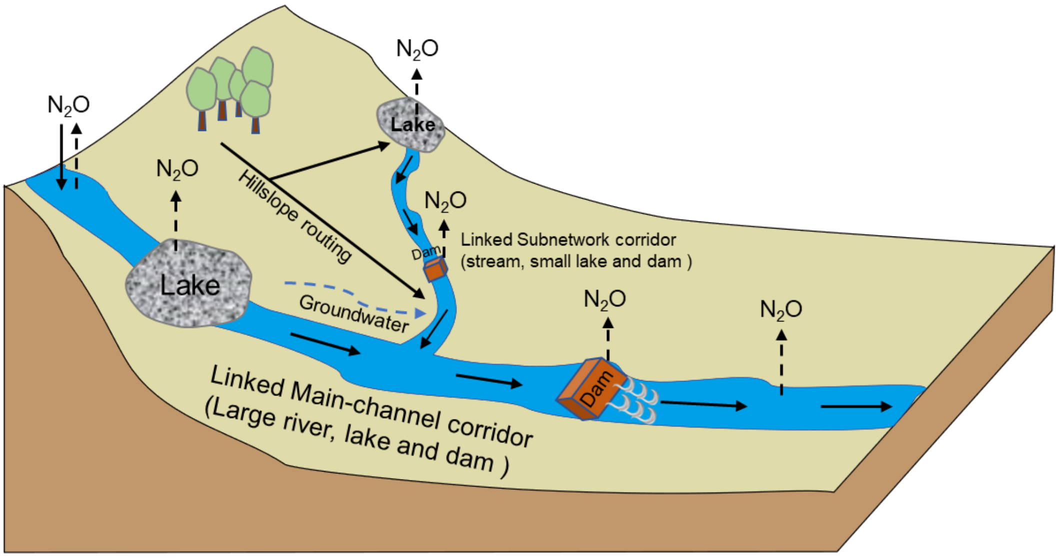

Supplementary Fig. 7: Concept model of water transport and N_2O emissions from inland waters.

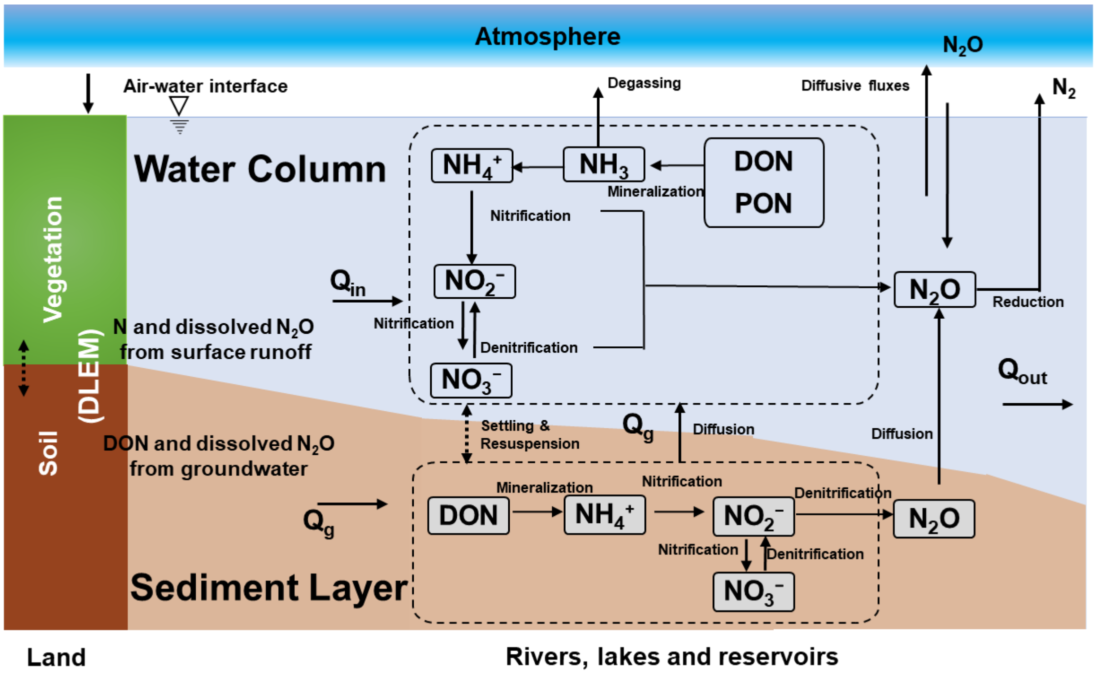

Supplementary Fig. 8: Concept model of N_2O biogeochemistry along the terrestrial aquatic continuum.

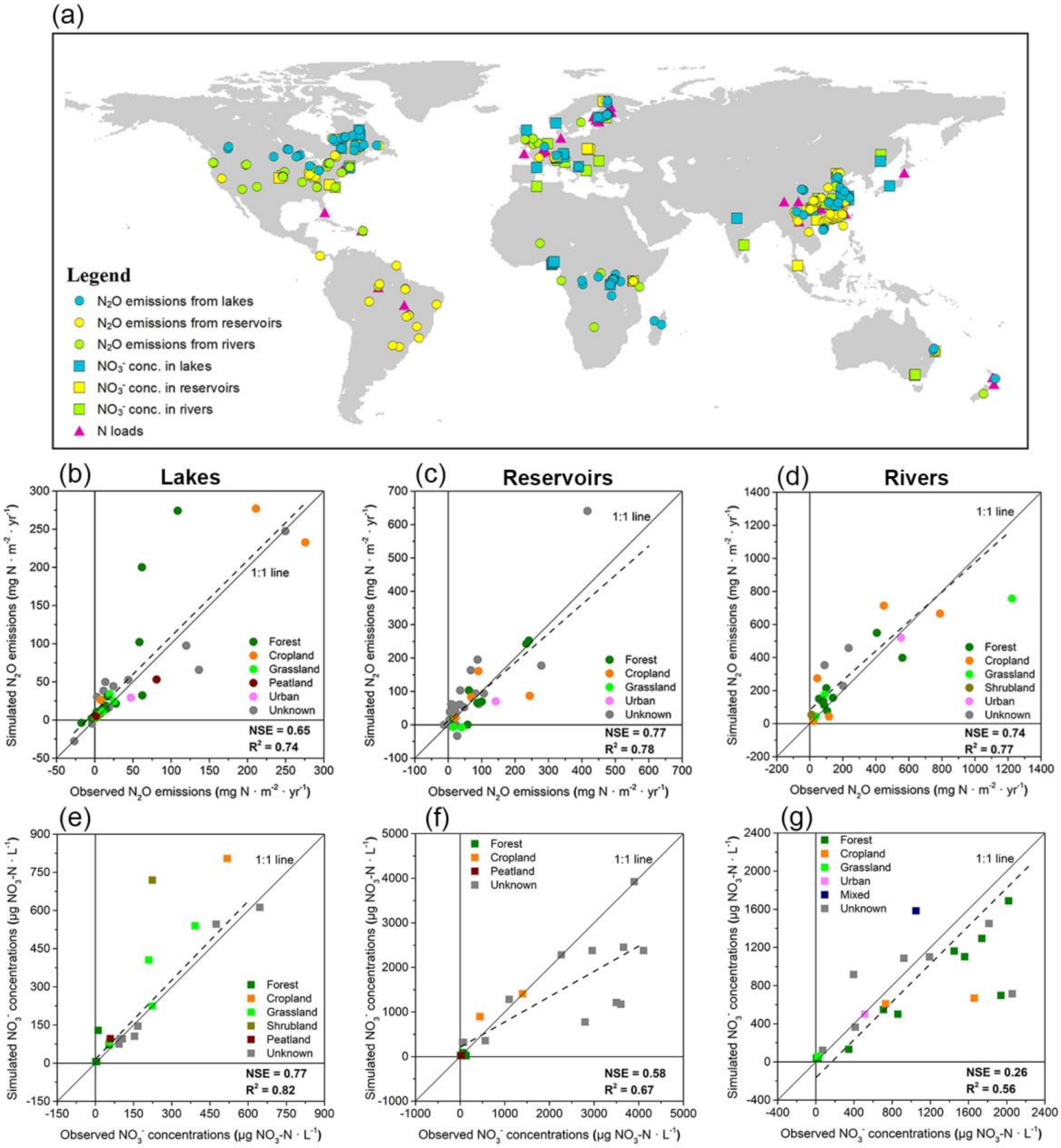

Supplementary Fig. 9: Comparisons of simulated inland water N_2O emissions and aquatic nitrate concentrations with observations. (a) The location map of observations. (b) (c) (d) The comparisons of simulated inland water N_2O emissions. (e) (f) (g) The comparisons of simulated aquatic nitrate concentrations. The sources of observed data used to validate inland water N_2O emissions and aquatic nitrate concentrations are provided in Tables S7 and S8. The map was drawn using the ArcMap v10.8.

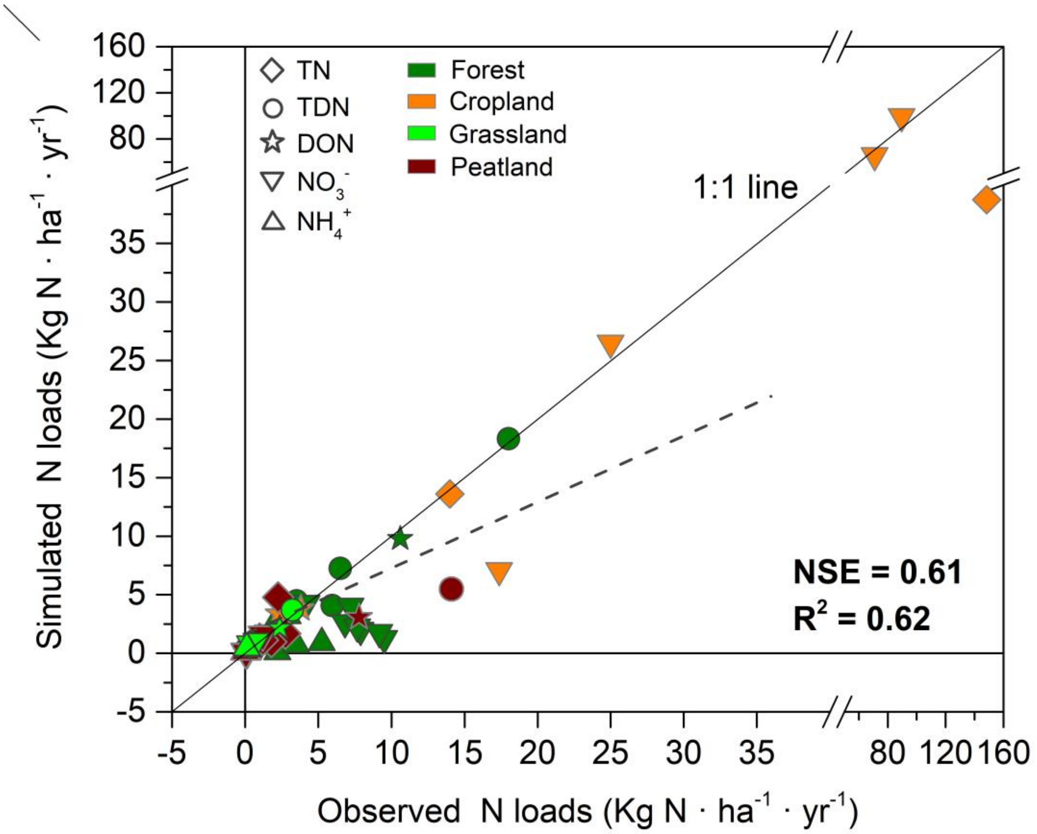

Supplementary Fig. 10: Comparisons of simulated terrestrial N loads with observations. The sources of observed data are provided in Tables S9.

Supplementary Reference:

1. Chow, V. T. A compendium of water-resources technology, Ven Te Chow. *Handbook of Applied Hydrology*), pp 8-61 (1964).
2. Biemans, H.*, et al.* Impact of reservoirs on river discharge and irrigation water supply during the 20th century. *Water Resources Research* **47**, (2011).
3. Lehner, B.*, et al.* High - resolution mapping of the world's reservoirs and dams for sustainable river - flow management. *Frontiers in Ecology and the Environment* **9**, 494-502 (2011).
4. Beaulieu, J. J.*, et al.* Nitrous oxide emission from denitrification in stream and river networks. *Proc. Natl. Acad. Sci. U. S. A.* **108**, 214-219 (2011).
5. Yang, Q.*, et al.* Increased nitrogen export from eastern North America to the Atlantic Ocean due to climatic and anthropogenic changes during 1901–2008. *Journal of Geophysical Research: Biogeosciences* **120**, 1046-1068 (2015).
6. Li, H.*, et al.* Evaluating global streamflow simulations by a physically based routing model coupled with the community land model. *Journal of Hydrometeorology* **16**, 948-971 (2015).
7. Getirana, A. C. V.*, et al.* The hydrological modeling and analysis platform (HyMAP): Evaluation in the Amazon basin. *Journal of Hydrometeorology* **13**, 1641-1665 (2012).
8. Raymond, P. A.*, et al.* Global carbon dioxide emissions from inland waters. *Nature* **503**, 355-359 (2013).
9. Sander, R. Compilation of Henry's law constants (version 4.0) for water as solvent. *Atmospheric Chemistry Physics* **15**, 4399-4981 (2015).
10. Fu, C., Lee, X., Griffis, T. J., Baker, J. M. & Turner, P. A. A modeling study of direct and indirect N2O emissions from a representative catchment in the US Corn Belt. *Water Resources Research* **54**, 3632-3653 (2018).
11. DelSontro, T., Beaulieu, J. J. & Downing, J. A. Greenhouse gas emissions from lakes and impoundments: upscaling in the face of global change. *Limnol. Oceanogr. Lett.* **3**, 64-75 (2018).
12. Zheng, Y.*, et al.* Global methane and nitrous oxide emissions from inland waters and estuaries. *Glob. Change Biol.* **28**, 4713–4725 (2022).
13. Lauerwald, R.*, et al.* Inland water greenhouse gas budgets for RECCAP2: 2. Regionalization and homogenization of estimates. *Glob. Biogeochem. Cycle* **37**, e2022GB007658 (2023).
14. Lauerwald, R.*, et al.* Natural lakes are a minor global source of N2O to the atmosphere. *Glob. Biogeochem. Cycle* **33**, 1564-1581 (2019).
15. Wang, J.*, et al.* Inland waters increasingly produce and emit nitrous oxide. *Environ. Sci. Technol.* **57**, 13506-13519 (2023).
16. Deemer, B. R.*, et al.* Greenhouse gas emissions from reservoir water surfaces: a new global synthesis. *BioScience* **66**, 949-964 (2016).
17. Maavara, T.*, et al.* Nitrous oxide emissions from inland waters: Are IPCC estimates too high? *Glob. Change Biol.* **25**, 473-488 (2019).
18. Soued, C., DelGiorgio, P. A. & Maranger, R. Nitrous oxide sinks and emissions in boreal aquatic networks in Québec. *Nature Geoscience* **9**, 116-120 (2016).
19. IPCC 2019 Refinement to the 2006 IPCC Guidelines for National Greenhouse Gas Inventories Volume 4 Agriculture, Forestry and Other Land Use. (2019).
20. Tian, L., Cai, Y. & Akiyama, H. A review of indirect N2O emission factors from agricultural nitrogen leaching and runoff to update of the default IPCC values. *Environ. Pollut.* **245**, 300-306 (2019).

21. Harrison, J. A., *et al.* The regional and global significance of nitrogen removal in lakes and reservoirs. *Biogeochemistry* **93**, 143-157 (2009).
22. Li, Q., Yu, Q., Wang, F., Yan, W. & Wang, J. Nitrogen removal in the Chaohu Lake, China: Implication in estimating lake N uptake velocity and modelling N removal efficiency of large lakes and reservoirs in the Changjiang River network. *Ecological Indicators* **124**, 107353 (2021).
23. McCrackin, M. L. & Elser, J. J. Atmospheric nitrogen deposition influences denitrification and nitrous oxide production in lakes. *Ecology* **91**, 528-539 (2010).
24. Klingensmith, K. M. & Alexander, V. Sediment nitrification, denitrification, and nitrous oxide production in a deep arctic lake. *Applied environmental microbiology* **46**, 1084-1092 (1983).
25. Goreau, T. J., *et al.* Production of NO2-and N2O by nitrifying bacteria at reduced concentrations of oxygen. *Applied Environmental Microbiology* **40**, 526-532 (1980).
26. Yao, Y., *et al.* Increased global nitrous oxide emissions from streams and rivers in the Anthropocene. *Nature Climate Change* **10**, 138-142 (2020).
27. Kolb, S. & Horn, M. A. Microbial CH4 and N2O consumption in acidic wetlands. *Frontiers in Microbiology* **3**, 78 (2012).
28. Borges, A. V., *et al.* Greenhouse gas emissions from African lakes are no longer a blind spot. *Sci. Adv.* **8**, eabi8716 (2022).
29. Huttunen, J. T., *et al.* Fluxes of methane, carbon dioxide and nitrous oxide in boreal lakes and potential anthropogenic effects on the aquatic greenhouse gas emissions. *Chemosphere* **52**, 609-621 (2003).
30. Miettinen, H., *et al.* Towards a more comprehensive understanding of lacustrine greenhouse gas dynamics—two-year measurements of concentrations and fluxes of CO2, CH4 and N2O in a typical boreal lake surrounded by managed forests. *Boreal Environment Research* **20**, 75-89 (2015).
31. Wang, H., Wang, W., Yin, C., Wang, Y. & Lu, J. Littoral zones as the “hotspots” of nitrous oxide (N2O) emission in a hyper-eutrophic lake in China. *Atmospheric Environment* **40**, 5522-5527 (2006).
32. Yang, Z., Zhao, Y. & Xia, X. Nitrous oxide emissions from Phragmites australis-dominated zones in a shallow lake. *Environ. Pollut.* **166**, 116-124 (2012).
33. Zhu, D., *et al.* Nitrous oxide emission from infralittoral zone and pelagic zone in a shallow lake: Implications for whole lake flux estimation and lake restoration. *Ecological Engineering* **82**, 368-375 (2015).
34. Li, Q., *et al.* Dominance of nitrous oxide production by nitrification and denitrification in the shallow Chaohu Lake, Eastern China: Insight from isotopic characteristics of dissolved nitrous oxide. *Environ. Pollut.* **255**, 113212 (2019).
35. Li, X. Study of greenhouse gas flux of water-air interface and its spatio-temporal change in Taihu Lake. Master of Engineering (Hohai University), (2005).
36. Zhao, J. Methane and nitrous oxide in the Changjiang and typical waters in eastern part of Hainan. (Ocean Univeristy of China, Qingdao), (2009).
37. Ma, W. W., Wang, H., Li, G., Zhao, J. M. & Wang, Y. S. A preliminary study of carbon dioxide, methane and nitrous oxide fluxes from the Gahai wetland. *Acta Prataculturae Sinica* **24**, 1-10 (2015).
38. Musenze, R. S., *et al.* Assessing the spatial and temporal variability of diffusive methane and nitrous oxide emissions from subtropical freshwater reservoirs. *Environ. Sci. Technol.* **48**,

14499-14507 (2014).
39. Downes, M. T. The production and consumption of nitrate in an eutrophic lake during early stratification. *Arch. Hydrobiol.* **122**, 257-274 (1991).
40. Salk, K. R. & Ostrom, N. E. Nitrous oxide in the Great Lakes: insights from two trophic extremes. *Biogeochemistry* **144**, 233-243 (2019).
41. Tangen, B. A., Finocchiaro, R. G., Gleason, R. A. & Dahl, C. F. Greenhouse gas fluxes of a shallow lake in south-central North Dakota, USA. *Wetlands* **36**, 779-787 (2016).
42. Salk, K. R.*, et al.* Ecosystem metabolism and greenhouse gas production in a mesotrophic northern temperate lake experiencing seasonal hypoxia. *Biogeochemistry* **131**, 303-319 (2016).
43. Tremblay, A. *Greenhouse gas emissions-fluxes and processes: hydroelectric reservoirs and natural environments* (Springer Science & Business Media) (2005).
44. Soued, C., Del Giorgio, P. A. & Maranger, R. Nitrous oxide sinks and emissions in boreal aquatic networks in Québec. *Nature Geoscience* **9**, 116-120 (2016).
45. Okuku, E. O., Bouillon, S., Tole, M. & Borges, A. V. Diffusive emissions of methane and nitrous oxide from a cascade of tropical hydropower reservoirs in Kenya. *Lakes Reservoirs: Research Management* **24**, 127-135 (2019).
46. Huttunen, J. T.*, et al.* Fluxes of CH4, CO2, and N2O in hydroelectric reservoirs Lokka and Porttipahta in the northern boreal zone in Finland. *Glob. Biogeochem. Cycle* **16**, 3-1-3-17 (2002).
47. Descloux, S., Chanudet, V., Serça, D. & Guérin, F. Methane and nitrous oxide annual emissions from an old eutrophic temperate reservoir. *Sci. Total Environ.* **598**, 959-972 (2017).
48. Wang, J., Wu, W., Zhou, X., Huang, Y. & Guo, M. Nitrous oxide (N2O) emissions from the high dam reservoir in longitudinal range-gorge regions on the Lancang-Mekong River, southwest China. *Journal of Environmental Management* **295**, 113027 (2021).
49. Liang, X.*, et al.* Control of the hydraulic load on nitrous oxide emissions from cascade reservoirs. *Environ. Sci. Technol.* **53**, 11745-11754 (2019).
50. Li, H., Yang, M., Zhang, M., Lei, T. & Lei, G. Characteristics and environmental determinants of grennhouse gas emissions from Yudushan Reservoir during growth season. *Chinese Journal of Ecology* **31**, 406-412 (2012).
51. Han, Y.*, et al.* Greenhouse gases emission characteristics of Naijing typical water in Spring. *China Environmental Science* **33**, 1360-1371 (2013).
52. Yang, L., Li, H. & Wang, J. Spatial and temporal variability of nitrous oxide emissions from a large subtropical Reservoir in Eastern China. *Polish Journal of Environmental Studies* **28**, (2019).
53. Zhang, X. Spatiotemporal variations of nitrous oxide (N2O) concentrations and flux in 17 reservoirs and their input rivers of Guangdong Province. (Jinan University, Guangzhou), (2012).
54. Xia, Y.*, et al.* Is indirect N2O emission a significant contributor to the agricultural greenhouse gas budget? A case study of a rice paddy-dominated agricultural watershed in eastern China. *Atmospheric Environment* **77**, 943-950 (2013).
55. Chen, N., Chen, Z., Wu, Y. & Hu, A. Understanding gaseous nitrogen removal through direct measurement of dissolved N2 and N2O in a subtropical river-reservoir system. *Ecological Engineering* **70**, 56-67 (2014).
56. Chen, J., Cao, W., Cao, D., Huang, Z. & Liang, Y. Nitrogen loading and nitrous oxide emissions from a river with multiple hydroelectric reservoirs. *Bulletin of Environmental Contamination Toxicology* **94**, 633-639 (2015).
57. Cheng, F.*, et al.* Distribution and emission of N2O in the largest river-reservoir system along the

Yellow River. *Sci. Total Environ.* **666**, 1209-1219 (2019).
58. Hao, Q., *et al.* Methane and nitrous oxide emissions from the drawdown areas of the Three Gorges Reservoir. *Sci. Total Environ.* **660**, 567-576 (2019).
59. Wang, X., *et al.* Greenhouse gases concentrations and fluxes from subtropical small reservoirs in relation with watershed urbanization. *Atmospheric Environment* **154**, 225-235 (2017).
60. Sturm, K., Yuan, Z., Gibbes, B., Werner, U. & Grinham, A. Methane and nitrous oxide sources and emissions in a subtropical freshwater reservoir, South East Queensland, Australia. *Biogeosciences* **11**, 5245-5258 (2014).
61. Jacinthe, P. A., Filippelli, G. M., Tedesco, L. P. & Raftis, R. Carbon storage and greenhouse gases emission from a fluvial reservoir in an agricultural landscape. *Catena* **94**, 53-63 (2012).
62. Guérin, F., Abril, G., Tremblay, A. & Delmas, R. Nitrous oxide emissions from tropical hydroelectric reservoirs. *Geophysical Research Letters* **35**, (2008).
63. Sikar, E., *et al.* Greenhouse gases and initial findings on the carbon circulation in two reservoirs and their watersheds. *Internationale Vereinigung für theoretische und angewandte Limnologie: Verhandlungen* **29**, 573-576 (2005).
64. dos Santos, M. A., *et al.* Estimates of GHG emissions by hydroelectric reservoirs: The Brazilian case. *Energy* **133**, 99-107 (2017).
65. Borges, A. V., *et al.* Globally significant greenhouse-gas emissions from African inland waters. *Nature Geoscience* **8**, 637-642 (2015).
66. Audet, J., Wallin, M. B., Kyllmar, K., Andersson, S. & Bishop, K. Nitrous oxide emissions from streams in a Swedish agricultural catchment. *Agriculture, Ecosystems & Environment* **236**, 295-303 (2017).
67. Dong, L. F., Nedwell, D. B., Colbeck, I. & Finch, J. Nitrous oxide emission from some English and Welsh rivers and estuaries. *Water, Air, Soil Pollution: Focus* **4**, 127-134 (2005).
68. Chen, N., Wu, J., Zhou, X., Chen, Z. & Lu, T. Riverine N2O production, emissions and export from a region dominated by agriculture in Southeast Asia (Jiulong River). *Agriculture, Ecosystems & Environment* **208**, 37-47 (2015).
69. Clough, T. J., Buckthought, L. E., Casciotti, K. L., Kelliher, F. M. & Jones, P. K. Nitrous oxide dynamics in a braided river system, New Zealand. *Journal of Environmental Quality* **40**, 1532-1541 (2011).
70. Beaulieu, J. J., Shuster, W. D. & Rebholz, J. A. Nitrous oxide emissions from a large, impounded river: The Ohio River. *Environ. Sci. Technol.* **44**, 7527-7533 (2010).
71. Cole, J. J. & Caraco, N. F. Emissions of nitrous oxide (N2O) from a tidal, freshwater river, the Hudson River, New York. *Environ. Sci. Technol.* **35**, 991-996 (2001).
72. Venkiteswaran, J. J., Rosamond, M. S. & Schiff, S. L. Nonlinear response of riverine N2O fluxes to oxygen and temperature. *Environ. Sci. Technol.* **48**, 1566-1573 (2014).
73. McMahon, P. B. & Dennehy, K. F. N2O emissions from a nitrogen-enriched river. *Environ. Sci. Technol.* **33**, 21-25 (1999).
74. Stow, C. A., Walker, J. T., Cardoch, L., Spence, P. & Geron, C. N2O emissions from streams in the Neuse River watershed, North Carolina. *Environ. Sci. Technol.* **39**, 6999-7004 (2005).
75. Beaulieu, J. J., *et al.* Nitrous oxide emission from denitrification in stream and river networks. *Proceedings of the National Academy of Sciences* **108**, 214-219 (2011).
76. Richey, J. E., Devol, A. H., Wofsy, S. C., Victoria, R. & Riberio, M. N. G. Biogenic gases and the oxidation and reduction of carbon in Amazon River and floodplain waters. *Limnology*

Oceanography **33**, 551-561 (1988).
77. Roland, F. A. E., Darchambeau, F., Morana, C. & Borges, A. V. Nitrous oxide and methane seasonal variability in the epilimnion of a large tropical meromictic lake (Lake Kivu, East-Africa). *Aquatic Sciences* **79**, 209-218 (2017).
78. Soja, G., Kitzler, B. & Soja, A. Emissions of greenhouse gases from Lake Neusiedl, a shallow steppe lake in Eastern Austria. *Hydrobiologia* **731**, 125-138 (2014).
79. Mosello, R*., et al.* Trends in the water chemistry of high altitude lakes in Europe. *Water, Air Soil Pollution: Focus* **2**, 75-89 (2002).
80. Xiao, Q*., et al.* Coregulation of nitrous oxide emissions by nitrogen and temperature in China's third largest freshwater lake (Lake Taihu). *Limnology Oceanography* **64**, 1070-1086 (2019).
81. Liu, L., Xu, M., Lin, M. & Zhang, X. Spatial variability of greenhouse gas effluxes and their controlling factors in the Poyang Lake in China. *Polish Journal of Environmental Studies* **22**, (2013).
82. Tamot, S. & Sharma, P. Physico-chemical status of upper lake (Bhopal, India) water quality with special reference to phosphate and nitrate concentration and their impact on lake ecosystem. *Asian J. Exp. Sci* **20**, 151-158 (2006).
83. Yoshida, M., Yoshida, T., Takashima, Y., Hosoda, N. & Hiroishi, S. Dynamics of microcystin-producing and non-microcystin-producing Microcystis populations is correlated with nitrate concentration in a Japanese lake. *FEMS Microbiology Letters* **266**, 49-53 (2007).
84. Yu, Q*., et al.* Tracking nitrate sources in the Chaohu Lake, China, using the nitrogen and oxygen isotopic approach. *Environmental Science Pollution Research* **25**, 19518-19529 (2018).
85. Liang, H., Zhai, D., Kong, X., Yuan, R. & Wang, S. Sources, migration and transformation of nitrate in Fuhe River and Baiyangdian Lake, China. *Chinese Journal of Eco-Agriculture* **25**, 1236-1244 (2017).
86. Jin, Z., Qin, X., Chen, L., Jin, M. & Li, F. Using dual isotopes to evaluate sources and transformations of nitrate in the West Lake watershed, eastern China. *Journal of Contaminant Hydrology* **177**, 64-75 (2015).
87. Wang, Z*., et al.* Nitrate dynamics during impoundment and flood periods in a subtropical karst reservoir: Hongfeng Lake, Southwestern China. *Environmental Science: Processes Impacts* **20**, 1736-1745 (2018).
88. Cao, Y., Tang, C., Song, X., Liu, C. & Zhang, Y. Characteristics of nitrate in major rivers and aquifers of the Sanjiang Plain, China. *Journal of Environmental Monitoring* **14**, 2624-2633 (2012).
89. Hemond, H. F. & Lin, K. Nitrate suppresses internal phosphorus loading in an eutrophic lake. *Water Research* **44**, 3645-3650 (2010).
90. Kijowska-Strugala, M., Wiejaczka, L. & Kozlowski, R. Influence of reservoirs on the concentration of nutrients in the water of mountain rivers. *Ecological Chemistry Engineering* **23**, 413-424 (2016).
91. Fadhullah, W*., et al.* Nitrate sources and processes in the surface water of a tropical reservoir by stable isotopes and mixing model. *Sci. Total Environ.* **700**, 134517 (2020).
92. Chen, S*., et al.* Seasonal variation of nitrogen biogeochemical processes constrained by nitrate dual isotopes in cascade reservoirs, Southwestern China. *Environmental Science Pollution Research* **28**, 26617-26627 (2021).
93. He, W., Chen, S., Liu, X. & Chen, J. Water quality monitoring in a slightly-polluted inland water

body through remote sensing—Case study of the Guanting Reservoir in Beijing, China. *Frontiers of Environmental Science Engineering in China* **2**, 163-171 (2008).
94. Beaulieu, J. J.*, et al.* Denitrification alternates between a source and sink of nitrous oxide in the hypolimnion of a thermally stratified reservoir. *Limnology Oceanography* **59**, 495-506 (2014).
95. Cunha, D. G. F., do Carmo Calijuri, M. & Dodds, W. K. Trends in nutrient and sediment retention in Great Plains reservoirs (USA). *Environmental Monitoring and Assessment* **186**, 1143-1155 (2014).
96. Mwanake, R. M.*, et al.* Land use, not stream order, controls N2O concentration and flux in the upper Mara River basin, Kenya. *Journal of Geophysical Research: Biogeosciences* **124**, 3491-3506 (2019).
97. Agency, European Environment Waterbase - Water Quality ICM, 2022. (2023).
98. Rajkumar, A. N., Barnes, J., Ramesh, R., Purvaja, R. & Upstill-Goddard, R. C. Methane and nitrous oxide fluxes in the polluted Adyar River and estuary, SE India. *Marine Pollution Bulletin* **56**, 2043-2051 (2008).
99. Qin, X.*, et al.* Assessment of indirect N2O emission factors from agricultural river networks based on long-term study at high temporal resolution. *Environ. Sci. Technol.* **53**, 10781-10791 (2019).
100. Wang, J., Chen, N., Yan, W., Wang, B. & Yang, L. Effect of dissolved oxygen and nitrogen on emission of N2O from rivers in China. *Atmospheric Environment* **103**, 347-356 (2015).
101. Giling, D. P., Grace, M. R., Thomson, J. R., Mac Nally, R. & Thompson, R. M. Effect of native vegetation loss on stream ecosystem processes: dissolved organic matter composition and export in agricultural landscapes. *Ecosystems* **17**, 82-95 (2014).
102. Schade, J. D., Bailio, J. & McDowell, W. H. Greenhouse gas flux from headwater streams in New Hampshire, USA: patterns and drivers. *Limnology Oceanography* **61**, S165-S174 (2016).
103. Salo, T. & Turtola, E. Nitrogen balance as an indicator of nitrogen leaching in Finland. *Agriculture, Ecosystems Environment* **113**, 98-107 (2006).
104. Muñoz-Carpena, R., Ritter, A., Bosch, D. D., Schaffer, B. & Potter, T. L. Summer cover crop impacts on soil percolation and nitrogen leaching from a winter corn field. *Agricultural Water Management* **95**, 633-644 (2008).
105. Long, G., Jiang, Y. & Sun, B. Seasonal and inter-annual variation of leaching of dissolved organic carbon and nitrogen under long-term manure application in an acidic clay soil in subtropical China. *Soil Tillage Research* **146**, 270-278 (2015).
106. Hussain, M. Z., Robertson, G. P., Basso, B. & Hamilton, S. K. Leaching losses of dissolved organic carbon and nitrogen from agricultural soils in the upper US Midwest. *Sci. Total Environ.* **734**, 139379 (2020).
107. Constantin, J.*, et al.* Effects of catch crops, no till and reduced nitrogen fertilization on nitrogen leaching and balance in three long-term experiments. *Agriculture, Ecosystem & Environment* **135**, 268-278 (2010).
108. Jankowski, K.*, et al.* Deep soils modify environmental consequences of increased nitrogen fertilizer use in intensifying Amazon agriculture. *Scientific Reports* **8**, 13478 (2018).
109. Yan, J., Li, K., Wang, W., Zhang, D. & Zhou, G. Changes in dissolved organic carbon and total dissolved nitrogen fluxes across subtropical forest ecosystems at different successional stages. *Water Resources Research* **51**, 3681-3694 (2015).
110. Ye, G. & Huang, B. Studies on geochemical cycling in Casuarina Equisetifolia plantation

ecosystems. *Journal of Nanjing Forestry University* **22**, 5-8 (1998).

111. Sha, L.*, et al.* Biogeochemical cycling of nitrogen at a tropical seasonal rain forest in Xishuangbanna, SW China. *Acta Phytoecologica Sinica* **26**, 689-694 (2002).

112. Chang, R.*, et al.* Nitrogen addition reduces dissolved organic carbon leaching in a montane forest. *Soil Biology Biochemistry* **127**, 31-38 (2018).

113. Xin, X. & Zhai, M. Studies on nutrition cycle of Abies georgei forest ecosystem of mountain Segila in Tibet. *Forest Research* **16**, 668-676 (2003).

114. Chen, Y., Lin, Y., Li, J., Liu, Y. & Yang, R. Rainfall process and nutrient dynamics of artificial Chinese fir plantation in Jiangxi Qianyanzhou experimental station. *Chinese Journal of Eco-Agriculture* **12**, 74-76 (2004).

115. You, Y.*, et al.* Hydrological fluxes of dissolved organic carbon and total dissolved nitrogen in subtropical forests at three restoration stages in southern China. *Journal of Hydrology* **583**, 124656 (2020).

116. Fang, Y., Gundersen, P., Mo, J. & Zhu, W Input and output of dissolved organic and inorganic nitrogen in subtropical forests of South China under high air pollution. *Biogeosciences* **5**, 339-352 (2008).

117. Hafner, S. D., Groffman, P. M. & Mitchell, M. J. Leaching of dissolved organic carbon, dissolved organic nitrogen, and other solutes from coarse woody debris and litter in a mixed forest in New York State. *Biogeochemistry* **74**, 257-282 (2005).

118. Kreibich, H., Lehmann, J., Scheufele, G. & Kern, J. Nitrogen availability and leaching during the terrestrial phase in a várzea forest of the Central Amazon floodplain. *Biology Fertility of Soils* **39**, 62-64 (2003).

119. Fang, Y.*, et al.* Large loss of dissolved organic nitrogen from nitrogen-saturated forests in subtropical China. *Ecosystems* **12**, 33-45 (2009).

120. Xu, Y., Zhou, G., Luo, T., Wu, Z. & He, Z. Soil solution chemistry and element budget in the forest ecosystem in Guangzhou. *Acta Ecologica Sinica* **21**, 1670-1681 (2001).

121. Zhang, X.*, et al.* Flux and distribution of some main ions in Karst forest in middle Guizhou Province. *Journal of Northeast Forestry University* **35**, 22-26 (2007).

122. Tian, D., Xiang, W. & Yang, W. Nutrient characteristics of hydrological process in young second rotation Chinese fir plantations. *Acta Ecologica Sinica* **22**, 859-865 (2002).

123. Wieder, R. K., Novák, M. & Cerný, J. *Biogeochemical Investigations at Watershed, Landscape, and Regional Scales: Refereed papers from BIOGEOMON, The Third International Symposium on Ecosystem Behavior; Co-Sponsored by Villanova University and the Czech Geological Survey; held at Villanova University, Villanova Pennsylvania, USA, June 21–25, 1997* (Springer Science & Business Media) (2013).

124. Templer, P. H., Silver, W. L., Pett-Ridge, J., M. DeAngelis, K. & Firestone, M. K. Plant and microbial controls on nitrogen retention and loss in a humid tropical forest. *Ecology* **89**, 3030-3040 (2008).

125. Scott, J. T.*, et al.* Carbon and nitrogen leaching under high and low phosphate fertility pasture with increasing nitrogen inputs. *Agriculture, Ecosystems and Environment* **202**, 139-147 (2015).

126. Buckthought, L. E., Clough, T. J., Cameron, K. C., Di, H. J. & Shepherd, M. A. Urine patch and fertiliser N interaction: Effects of fertiliser rate and season of urine application on nitrate leaching and pasture N uptake. *Agriculture, Ecosystems and Environment* **203**, 19-28 (2015).

127. Wachendorf, C., Taube, F. & Wachendorf, M. Nitrogen leaching from 15 N labelled cow urine and dung applied to grassland on a sandy soil. *Nutrient Cycling in Agroecosystems* **73**, 89-100

(2005).

128. Koskinen, M., Sallantaus, T. & Vasander, H. Post-restoration development of organic carbon and nutrient leaching from two ecohydrologically different peatland sites. *Ecological Engineering* **37**, 1008-1016 (2011).

129. Edokpa, D. A., Evans, M. G. & Rothwell, J. J. High fluvial export of dissolved organic nitrogen from a peatland catchment with elevated inorganic nitrogen deposition. *Sci. Total Environ.* **532**, 711-722 (2015).

130. Kortelainen, P*., et al.* Controls on the export of C, N, P and Fe from undisturbed boreal catchments, Finland. *Aquatic Sciences* **68**, 453-468 (2006).
